# Optimal dosing regimen of ondansetron oral soluble pellicles for preventing postoperative nausea and vomiting after primary total joint arthroplasty: a randomized controlled clinical trial

**DOI:** 10.3389/fmed.2025.1702879

**Published:** 2025-12-04

**Authors:** Yinghao Wang, Shangjie Zhong, Long Zhao, Zongke Zhou, Haoyang Wang

**Affiliations:** 1 Department of Orthopedics and Research Institute of Orthopedics, West China Hospital, Sichuan University, Chengdu, China; 2 Department of Orthopedics, West China Hospital, Sichuan University, Chengdu, China

**Keywords:** total joint arthroplasty, postoperative nausea and vomiting, ondansetron oral soluble pellicles, optimal dosing, clinical significance

## Abstract

**Background:**

The widespread use of high-dose antifibrinolytic agents (tranexamic acid) and opioids in total joint arthroplasty (TJA) has highlighted a critical concern: postoperative nausea and vomiting (PONV). Ondansetron, recognized as a cornerstone medication for PONV prevention in clinical guidelines, offers an innovative solution through its oral soluble pellicle (OSP) formulation. We conducted this study to investigate the optimal dosing regimen of ondansetron OSPs for preventing PONV following primary TJA.

**Methods:**

This randomized, controlled, double-masked clinical trial allocated 198 patients to three intervention groups: (1) Preop 16 mg group: Two ondansetron OSPs (16 mg) administered orally 1 h before induction, followed by placebo films at 6 and 12 h postoperatively; (2) 16 + 8 mg group: Two ondansetron OSPs (16 mg) administered preoperatively, followed by one ondansetron OSP (8 mg) at 6 h and one placebo film at 12 h postoperatively; (3) 8 + 8 + 8 mg group: One ondansetron OSP (8 mg) and one placebo film administered preoperatively, followed by one ondansetron OSP (8 mg) at both 6 and 12 h postoperatively. The primary outcome assessed the incidence and severity of PONV within 48 h after TJA. Secondary outcomes included the utilization frequency of rescue metoclopramide and tramadol, along with adverse drug reactions (ADRs).

**Results:**

The primary outcomes demonstrated that both the Preop 16 mg and 16 + 8 mg groups achieved significantly lower incidences of postoperative nausea compared to the 8 + 8 + 8 mg group, although no statistically significant differences were observed among the three groups in nausea severity (visual analog scale (VAS) scores) or postoperative vomiting frequency. The secondary outcomes showed significantly reduced metoclopramide utilization in the 16 + 8 mg group versus the 8 + 8 + 8 mg group, with a non-significant trend in the Preop 16 mg group, while tramadol usage and ADR rates showed no significant intergroup differences.

**Conclusion:**

Compared to the 8 + 8 + 8 mg regimen, both the Preop 16 mg and 16 + 8 mg dosing strategies demonstrated superior efficacy. For high-risk patients, the 16 + 8 mg regimen may represent the optimal therapeutic approach.

## Introduction

End-stage hip and knee disorders cause debilitating pain and functional limitations, especially among geriatric patients, which are effectively alleviated through total joint arthroplasty (TJA) (1). In recent years, the promotion of Enhanced Recovery After Surgery (ERAS) principles has progressively refined perioperative management protocols for TJA. While high-dose antifibrinolytic agents (tranexamic acid) and opioids are widely used to reduce bleeding and alleviate pain (2–4), they concurrently trigger postoperative nausea and vomiting (PONV)—a complication severely impairing surgical experiences (5–7). Studies indicate that PONV affects approximately 30% of patients perioperatively, with the incidence soaring to 80% in high-risk cohorts lacking preventive measures (8–10). PONV can lead to restricted oral intake, aspiration risk, electrolyte imbalances, and wound dehiscence (11), all of which critically undermine rehabilitation. To optimize perioperative management in TJA, the prevention of PONV demands prioritized attention and systematic intervention.

Ondansetron is recognized as a cornerstone medication for PONV prophylaxis in international guidelines (8, 12, 13). As a potent and highly selective serotonin 5-HT3 receptor antagonist, it inhibits the emetic reflex by blocking peripheral and central 5-HT3 receptors (14, 15). Available formulations include oral tablets, orally disintegrating tablets, injectable solutions, and oral soluble pellicles (OSPs). The OSP formulation adheres to the oral mucosa via saliva, dissolving within seconds to deliver the drug directly into systemic circulation through submucosal vessels. This delivery system eliminates the first-pass effect inherent to conventional oral tablets and avoids risks associated with intravenous administration (e.g., infection, bleeding) (16, 17). Studies by Sun and Hegde et al. have validated the efficacy and safety of ondansetron OSPs for preventing chemotherapy-induced nausea/vomiting and PONV following laparoscopic surgeries (18, 19).

Our preliminary data (unpublished) further demonstrate its effectiveness in PONV prevention after TJA. To establish the optimal dosing regimen for ondansetron OSPs in patients undergoing TJA, we conducted this randomized controlled trial.

## Methods

This randomized, controlled, double-masked clinical trial was conducted from May to July 2025. The study protocol received approval from the Institutional Review Board (Approval No. 2071; 2024) and was prospectively registered with the Chinese Clinical Trial Registry (Registration ID: ChiCTR2500097588). Written informed consent was obtained from all participants prior to enrollment.

### Patient selection

This study enrolled adult patients undergoing primary unilateral TJA for end-stage hip or knee disorders. The exclusion criteria comprised the following: (1) Documented hypersensitivity to ondansetron; (2) preoperative use of corticosteroids, antiemetics, or chemotherapeutic agents; (3) comorbidities potentially inducing nausea/vomiting (e.g., gastrointestinal diseases, Ménière’s disease); (4) suboptimal perioperative glycemic or blood pressure control; and (5) concurrent participation in other clinical trials or anticipated poor protocol adherence.

### Randomization and blinding

The allocation sequence was computer-generated using blocked randomization by Investigator 1 and concealed in opaque, sealed envelopes. Immediately prior to surgery, Investigator 2 opened one envelope to disclose group assignment and administered the corresponding study medication or placebo. Investigator 3 assessed and recorded outcome measures, with anonymized data subsequently transferred to Investigator 4 for statistical analysis. All investigators worked independently, with capped access to information strictly necessary for their designated roles. Patients, surgeons, anesthesiologists, and nursing staff remained blinded to group assignment throughout the trial.

### Perioperative management

All patients received a standardized ERAS perioperative protocol. Preemptive analgesia was provided with oral imrecoxib (100 mg twice daily), starting 1 day before surgery. Solid food intake was prohibited for 6 h preoperatively, with clear liquids restricted for 2 h prior to anesthesia induction.

All patients received a combined intravenous-inhalational anesthesia protocol comprising induction with sufentanil (0.4 μg/kg), cisatracurium (0.2 mg/kg), ciprofol (0.4 mg/kg), and midazolam (0.2 mg/kg), followed by maintenance with remifentanil infusion (0.15–0.2 μg/kg/min) and sevoflurane (1–3% end-tidal concentration), supplemented with additional cisatracurium and sufentanil boluses based on surgical duration. Throughout anesthesia administration, corticosteroids (except for allergy rescue) and intravenous antiemetics, including ondansetron, were strictly prohibited.

For total hip arthroplasty, a posterolateral approach was utilized, while total knee arthroplasty employed a midline incision with medial parapatellar arthrotomy. Tranexamic acid (2 g) was administered intravenously 10 min prior to skin incision. All procedures were performed without tourniquet application, with intraoperative hemostasis maintained through electrocautery and conventional techniques. Prior to wound closure, periarticular infiltration analgesia with ropivacaine (200 mg) was administered. Surgical drains were not used in any patients.

Postoperatively, analgesia was maintained with oral imrecoxib (100 mg twice daily) and topical flurbiprofen gel patches (40 mg twice daily), supplemented with rescue tramadol (100 mg IV) for pain scores >5 or upon patient request. Tranexamic acid (1 g IV) was administered at 3, 6, 12, and 24 h for hemorrhage control.

The patients were randomized to three ondansetron OSP regimens: The Preop 16 mg group (two 8 mg OSPs 1 h pre-induction + one placebo at 6 h and 12 h), the 16 + 8 mg group (two 8 mg OSPs 1 h pre-induction + one 8 mg OSP at 6 h + one placebo at 12 h), and the 8 + 8 + 8 mg group (one 8 mg OSP + one placebo 1 h pre-induction + one 8 mg OSP at both 6 h and 12 h). Rescue metoclopramide (10 mg IV) was administered for nausea visual analog scale (VAS) scores >5, emesis, or upon patient request.

### Outcome measures

#### Primary outcomes

PONV was recorded and analyzed using two distinct subcategories: postoperative nausea (PON) and postoperative vomiting (POV). PON was defined as the conscious perception of an urge to vomit or retch, with documentation encompassing both episode frequency and severity quantified via a validated 0–10 Visual Analog Scale (VAS), where 0 represented “no nausea” and 10 signified “nausea as bad as it possibly could be.” POV was defined as the forceful expulsion of gastric contents through the oral cavity. Assessments were conducted every 3 h during the first 6 postoperative h, followed by cumulative evaluations over the 7–24 h and 24–48 h intervals.

#### Secondary outcomes

Secondary outcomes included the frequency of postoperative tramadol and metoclopramide administration, along with ondansetron-related adverse drug reactions (ADRs) within 48 h after surgery, including headache, dizziness, and cutaneous flushing.

### Data analysis

The sample size calculation was based on prior literature, which indicated an anticipated 30% reduction in PONV incidence with ondansetron compared to placebo, projecting a 20% incidence rate in the ondansetron group versus 50% in the placebo group (19, 20). With 90% statistical power and a two-sided alpha level of 0.05, a minimum of 66 patients per group was required. To account for potential attrition and missing data, 215 participants were enrolled.

Continuous variables were expressed as mean ± standard deviation. For normally distributed continuous variables with homogeneity of variance, one-way ANOVA was applied; otherwise, the Kruskal–Wallis test was used. Categorical variables were presented as counts and percentages, analyzed using chi-squared or Fisher’s exact tests. Pairwise comparisons among the three groups were performed using the Bonferroni correction (adjusted significance level: 0.05/3 ≈ 0.017). Kaplan–Meier survival curves were generated, and between-group differences were evaluated using a log-rank test. All analyses were performed using IBM SPSS Statistics (Version 23.0), with statistical significance defined as a *p*-value of < 0.05.

## Results

**Figure 1:**
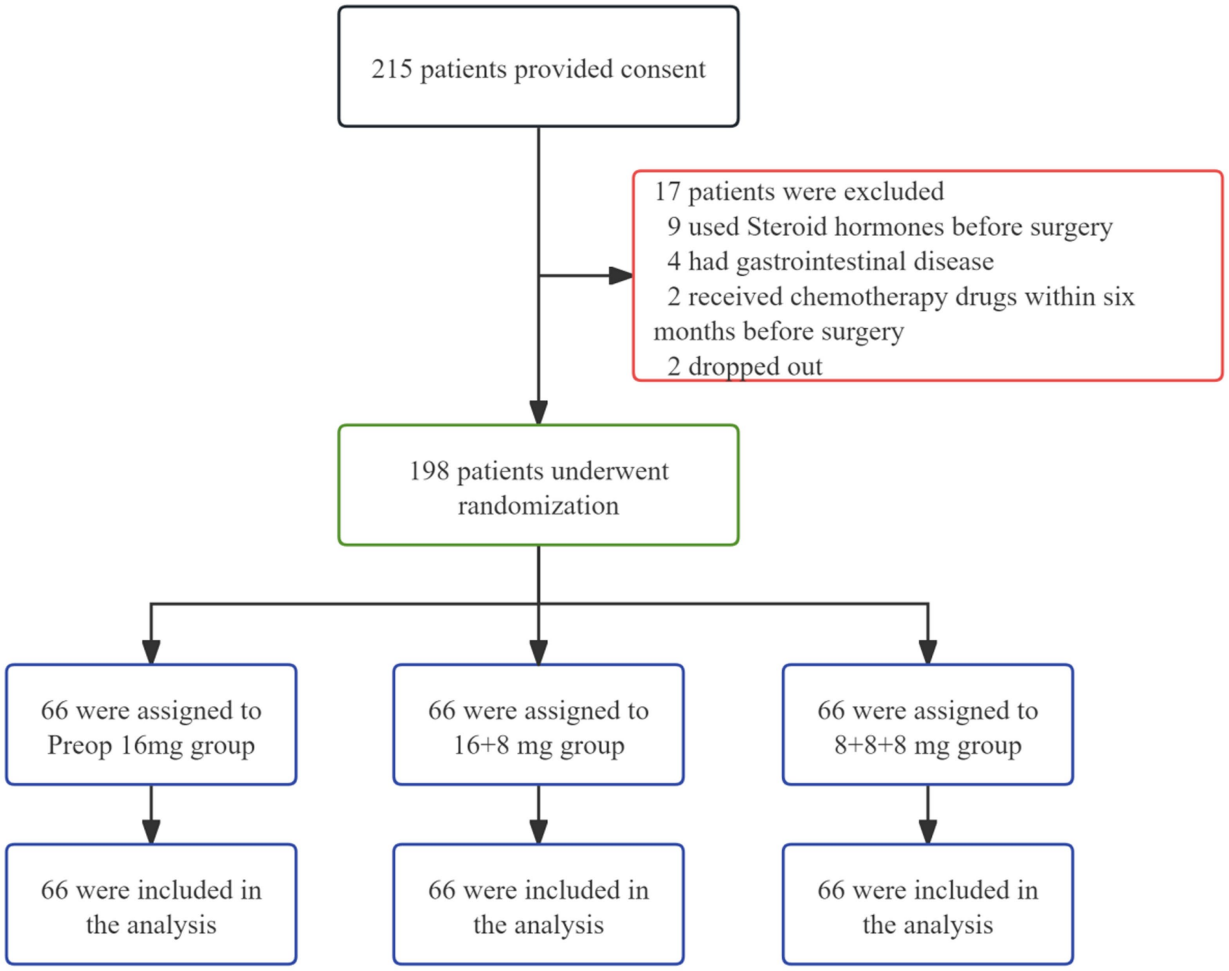
Flowchart of participant enrollment and allocation.

In this study, informed consent was obtained from 215 patients, of whom 17 withdrew during the trial. The remaining 198 participants were randomly allocated to three groups: The Preop 16 mg group, the 16 + 8 mg group, and the 8 + 8 + 8 mg group (Figure 1). Baseline characteristics across all groups showed no statistically significant differences (Table 1).

**Table 1:** Baseline characteristics.

Characteristic	Preop 16 mg (*N* = 66)	16 + 8 mg (*N* = 66)	8 + 8 + 8 mg (*N* = 66)	*p*-value
Age (years)	60.95 ± 11.06	61.50 ± 11.85	62.23 ± 12.40	0.824
Body Mass Index (kg/m^2^)	24.39 ± 3.31	24.72 ± 3.71	24.43 ± 3.70	0.847
Female sex (%)	40 (60.6)	46 (69.7)	46 (69.7)	0.441
Surgical site (knee %)	29 (43.9)	27 (40.9)	32 (48.5)	0.678
Surgical duration (mins)	80.70 ± 20.75	82.52 ± 26.54	82.02 ± 26.10	0.908
Anesthesia duration (mins)	118.67 ± 21.50	119.70 ± 28.31	119.47 ± 26.48	0.971
ASA (II %)	51 (77.3)	53 (80.3)	49 (74.2)	0.630
History of PONV (%)	7 (10.6)	8 (12.1)	7 (10.6)	0.950
History of smoking (%)	18 (27.3)	10 (15.2)	13 (19.7)	0.222
History of motion sickness (%)	18 (27.3)	21 (31.8)	23 (34.8)	0.640

ASA, American Society of Anesthesiologists; PONV, postoperative nausea and vomiting.

### Primary outcomes

#### PON

During the short-term postoperative intervals (0–3, 3–6, 6–24, and 24–48 h), no statistically significant differences in PON incidence were observed among the three groups (*p* > 0.05). However, during the 0–3 and 3–6 h intervals, both the Preop 16 mg group and the 16 + 8 mg group demonstrated an absolute risk reduction (ARR) exceeding 9% compared to the 8 + 8 + 8 mg group. The ARR was calculated as the arithmetic difference in PON incidence between the groups.

During the extended observation period (0–6 h), the 16 + 8 mg group exhibited a significantly lower incidence (15.2%) compared to the 8 + 8 + 8 mg group (33.3%), with P3† (16 + 8 mg vs. 8 + 8 + 8 mg) < 0.017. Over the 0–24 and 0–48 h intervals, the 8 + 8 + 8 mg group demonstrated significantly higher PON incidence (36.4 and 37.9%, respectively) than both the Preop 16 mg group (16.7 and 18.2%) and the 16 + 8 mg group (15.2 and 15.2%), as evidenced by P2† (Preop 16 mg vs. 8 + 8 + 8 mg) and P3† < 0.017.

The Preop 16 mg and 16 + 8 mg groups exhibited no statistically significant differences in the incidence of PON, all P1† (Preop 16 mg vs. 16 + 8 mg) > 0.017 (Table 2).

**Table 2:** Incidence of nausea.

Time	Preop 16 mg (*N* = 66)	16 + 8 mg (*N* = 66)	8 + 8 + 8 mg (*N* = 66)	P* value	P1†	P2†	P3†
0–3 h	7 (10.6)	6 (9.1)	14 (21.2)	0.087			
3–6 h	6 (9.1)	6 (9.1)	12 (18.2)	0.181			
0–6 h	12 (18.2)	11 (16.7)	22 (33.3)	**0.041**	0.819	0.047	0.027
6–24 h	2 (3.0)	3 (4.5)	3 (4.5)	0.878			
0–24 h	12 (18.2)	11 (16.7)	24 (36.4)	**0.013**	0.819	0.019	**0.010**
24–48 h	3 (4.5)	3 (4.5)	3 (4.5)	1.000			
0–48 h	12 (18.2)	12 (18.2)	25 (37.9)	**0.010**	1.000	**0.012**	**0.012**

* Uncorrected *p*-values. † Bonferroni-corrected *p*-values (0.05/3 = 0.017): P1, Preop 16 mg vs. 16 + 8 mg; P2, Preop 16 mg vs. 8 + 8 + 8 mg; P3, 16 + 8 mg vs. 8 + 8 + 8 mg. The bold *p*-values means significant.

Similarly, VAS scores showed no significant intergroup differences among the three groups, with P1†, P2†, P3† > 0.017 (Table 3).

**Table 3:** Visual analog scale severity for nausea.

Time	Preop 16 mg (*N* = 66)	16 + 8 mg (*N* = 66)	8 + 8 + 8 mg (*N* = 66)	P* value	P1†	P2†	P3†
0–3 h	0.59 ± 1.98	0.76 ± 2.34	1.59 ± 3.24	0.060			
3–6 h	0.62 ± 2.02	0.59 ± 1.94	1.58 ± 3.48	**0.045**	1.000	0.081	0.194
6–24 h	0.21 ± 1.01	0.18 ± 1.04	0.38 ± 1.82	0.664			
24–48 h	0.30 ± 1.43	0.21 ± 1.04	0.36 ± 1.69	0.825			

* Uncorrected *p*-values. † Bonferroni-corrected *p*-values (0.05/3 = 0.017): P1, Preop 16 mg vs. 16 + 8 mg; P2, Preop 16 mg vs. 8 + 8 + 8 mg; P3, 16 + 8 mg vs. 8 + 8 + 8 mg. The bold *p*-values means significant.

**Figure 2:**
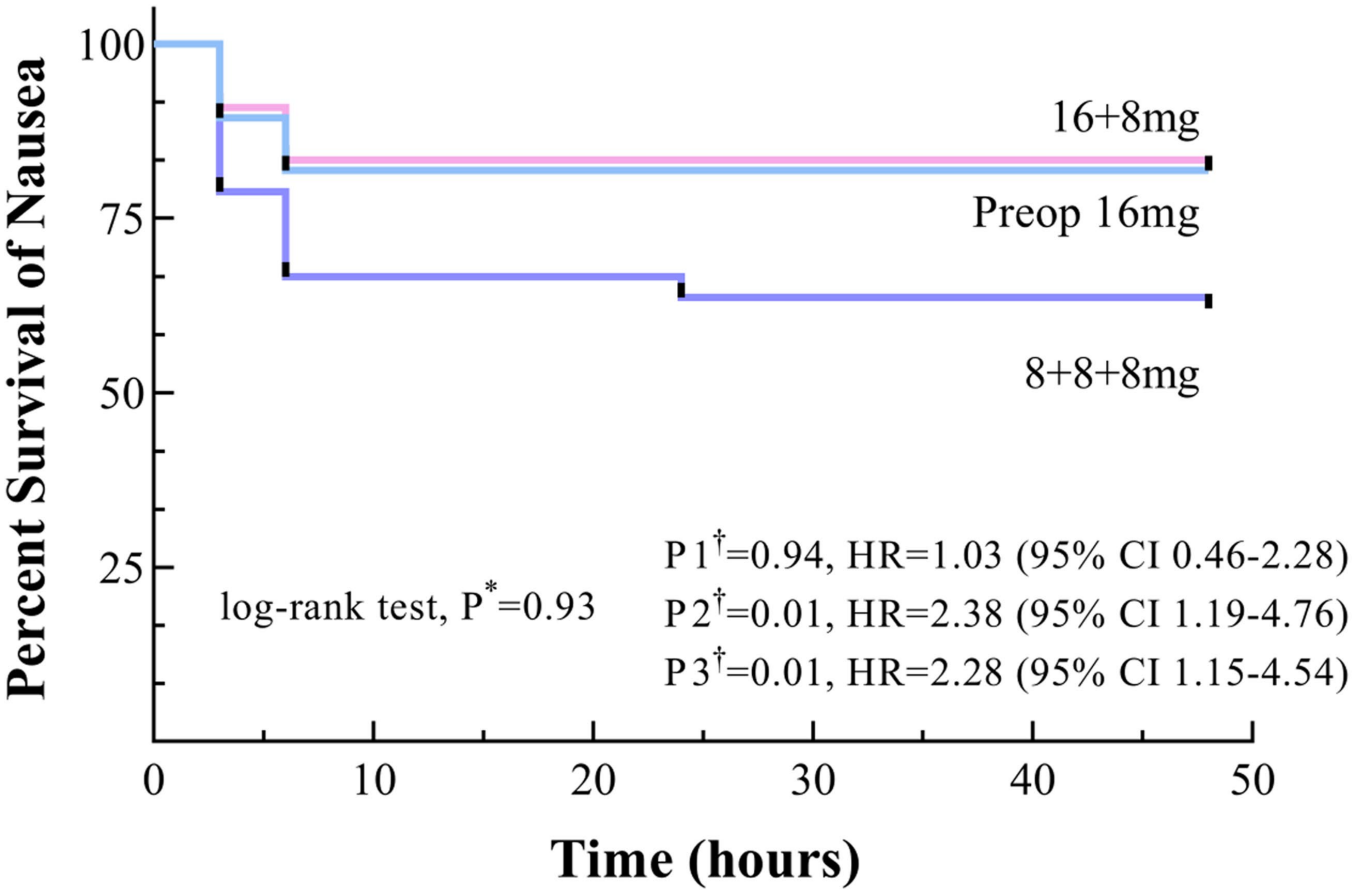
Time to onset of nausea within 48 h postoperatively, shown as Kaplan–Meier curves. * Uncorrected *p* values. †Bonferroni-corrected *p* values (0.05/3 = 0.017): P1, Preop 16 mg versus 16 + 8 mg; P2, Preop 16 mg versus 8 + 8 + 8 mg; and P3, 16 + 8 mg versus 8 + 8 + 8 mg.

Kaplan–Meier survival curves for the Preop 16 mg and 16 + 8 mg groups consistently trended above the 8 + 8 + 8 mg group throughout the observation period, although log-rank testing indicated no statistically significant difference (*p* = 0.93). The 48-h PONV-free survival rates were 81.8% for the Preop 16 mg group and 84.8% for the 16 + 8 mg group, both significantly higher than that of the 8 + 8 + 8 mg group (62.1%), with hazard ratios (HR) of 2.38 (95% CI 1.19–4.76) and 2.28 (95% CI 1.15–4.54), respectively (P2† & P3† < 0.017). Critically, survival curve comparisons between the Preop 16 mg and 16 + 8 mg groups revealed no significant difference, P1† = 0.94 (Figure 2).

#### POV

The incidence of POV showed no statistically significant differences among the three groups, all P* > 0.05 (Table 4).

**Table 4:** Incidence of vomiting.

Time	Preop 16 mg (*N* = 66)	16 + 8 mg (*N* = 66)	8 + 8 + 8 mg (*N* = 66)	P* value	P1†	P2†	P3†
0–3 h	5 (7.6)	6 (9.1)	11 (16.7)	0.205			
3–6 h	3 (4.5)	5 (7.6)	8 (12.1)	0.275			
0–6 h	7 (10.6)	9 (13.6)	12 (18.2)	0.454			
6–24 h	2 (3.0)	1 (1.5)	2 (3.0)	0.814			
0–24 h	9 (13.6)	9 (13.6)	14 (21.2)	0.394			
24–48 h	2 (3.0)	3 (4.5)	2 (3.0)	0.862			
0–48 h	10 (15.2)	11 (16.7)	14 (21.2)	0.637			

* Uncorrected *p*-values. † Bonferroni-corrected *p*-values (0.05/3 = 0.017): P1, Preop 16 mg versus 16 + 8 mg; P2, Preop 16 mg versus 8 + 8 + 8 mg; and P3, 16 + 8 mg versus 8 + 8 + 8 mg.

**Figure 3:**
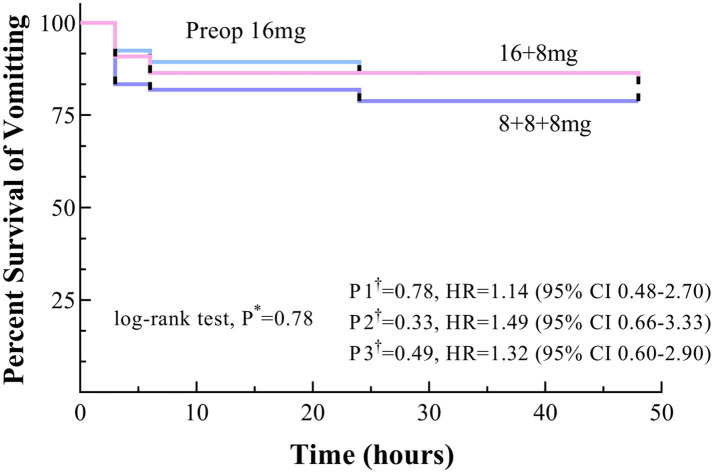
Time to onset of vomiting within 48 h postoperatively, shown as Kaplan–Meier curves. * Uncorrected *p* values. †Bonferroni-corrected *p* values (0.05/3 = 0.017): P1, Preop 16 mg versus 16 + 8 mg; P2, Preop 16 mg versus 8 + 8 + 8 mg; and P3, 16 + 8 mg versus 8 + 8 + 8 mg.

Kaplan–Meier survival curves for the Preop 16 mg and 16 + 8 mg groups consistently trended above the 8 + 8 + 8 mg group throughout the observation period, although no significant differences were observed, with P* > 0.05, P1†, P2†, P3† > 0.017 (Figure 3).

### Secondary outcomes

The 16 + 8 mg group demonstrated a significantly lower metoclopramide utilization frequency (7.6%) compared to the 8 + 8 + 8 mg group (22.7%), with P3† < 0.017. No statistically significant differences in tramadol usage rates were observed among the three groups (P* > 0.05).

Adverse drug reactions across all groups were predominantly limited to headache, dizziness, and cutaneous flushing, with incidences below 5% and no significant intergroup differences (Table 5).

**Table 5:** Comparison of secondary outcomes.

Secondary outcomes	Preop 16 mg (*N* = 66)	16 + 8 mg (*N* = 66)	8 + 8 + 8 mg (*N* = 66)	P* value	P1^†^	P2^†^	P3^†^
Metoclopramide	6 (9.1)	5 (7.6)	15 (22.7)	**0.018**	0.753	0.032	**0.015**
Tramadol	10 (15.2)	8 (12.1)	9 (13.6)	0.879			
Adverse drug reactions	5 (7.6)	4 (6.1)	4 (6.1)	0.921			
Headache	1 (1.5)	0	2 (3.0)	0.362			
Dizziness	3 (4.5)	3 (4.5)	2 (3.0)	0.878			
Cutaneous flushing	1 (1.5)	1 (1.5)	0	0.603			

* Uncorrected *p*-values. † Bonferroni-corrected *p*-values (0.05/3 = 0.017): P1, Preop 16 mg vs. 16 + 8 mg; P2, Preop 16 mg vs. 8 + 8 + 8 mg; P3, 16 + 8 mg vs. 8 + 8 + 8 mg. The bold *p*-values means significant.

## Discussion

Our previous study (unpublished data) established the efficacy and safety of preoperative 16 mg ondansetron OSP for preventing PONV following TJA. We observed that while the peak incidence of PONV occurs within the first 6 postoperative h (21, 22), a subset of patients continues to experience PONV beyond this critical window, which significantly impedes early postoperative recovery. To further reduce perioperative PONV incidence in TJA, we conducted this trial to determine the optimal dosing regimen for ondansetron OSP prophylaxis. The results demonstrated no statistically significant differences between the Preop 16 mg and 16 + 8 mg regimens, with both proving superior to the 8 + 8 + 8 mg dosing strategy.

During the 0–6 h postoperative interval, the Preop 16 mg group exhibited a 16.6% reduction in PON incidence compared to the 8 + 8 + 8 mg group (16.7% vs. 33.3%), suggesting superior prophylactic efficacy of the Preop 16 mg regimen. However, this difference did not reach statistical significance (P2† > 0.017). Both the Preop 16 mg and 16 + 8 mg groups consistently demonstrated lower nausea VAS scores than the 8 + 8 + 8 mg group across all observation periods. For POV, ARR values exceeding 7% were observed for both the Preop 16 mg and 16 + 8 mg groups compared to the 8 + 8 + 8 mg group during the 0–6 h and 0–24 h intervals, although statistical significance was not achieved. We attribute this statistical limitation to the original sample size calculation, which was powered to detect a 30% absolute reduction in PONV incidence—a threshold substantially larger than the observed intergroup differences (<30%). Consequently, the study lacked sufficient statistical power to confirm the clinical superiority of the Preop 16 mg and 16 + 8 mg regimens at the prespecified significance level. Nevertheless, based on the direction and magnitude of risk reduction, both the Preop 16 mg and 16 + 8 mg regimens demonstrated clinically meaningful advantages over the fragmented dosing regimen (8 + 8 + 8 mg) for preventing PONV following TJA.

We observed that ADRs associated with ondansetron OSP primarily manifested as headache, dizziness, and cutaneous flushing, consistent with patterns documented in prior literature (23, 24). The incidence of these ADRs remained below 5% across all dosing groups, with no statistically significant differences, confirming the favorable safety profile of ondansetron OSP. Compared to the 8 + 8 + 8 mg group, the Preop 16 mg group demonstrated an ARR value of 13.6% in metoclopramide utilization. Although statistical significance was not achieved, this clinically meaningful reduction supports the efficacy of the Preop 16 mg regimen in reducing rescue antiemetic demand.

Currently validated risk factors for PONV in adults include female sex, a history of PONV or motion sickness, non-smoking status, younger age, postoperative opioid use, and general anesthesia (8, 10, 25, 26). The Apfel score stratifies risk based on four variables: female sex, a history of PONV/motion sickness, non-smoking status, and postoperative opioid use. PONV incidence correlates with Apfel scores as follows: 10% (0 factors), 20% (1), 40% (2), 60% (3), and 80% (4) (10). The Koivuranta score further incorporates surgical duration >60 min as an additional risk variable (26). These findings suggest that although no significant difference exists between the Preop 16 mg and 16 + 8 mg regimens for general PONV prophylaxis, the 16 + 8 mg regimen is recommended for high-risk patients (Apfel score ≥ 3 or prolonged surgeries) to ensure sustained therapeutic coverage against delayed-phase emesis.

In our study, we used the term “clinically meaningful.” We therefore discuss the distinction between statistical significance and clinical significance. Statistical significance is determined by the *p*-value and confidence intervals. Typically, when a difference with a p-value < 0.05 is found, it is termed “statistically significant.” Statistical significance indicates that the difference is unlikely to be due to chance or sampling error. Clinical significance refers to an outcome that improves the patient’s quality of life and makes them feel well and function well. Clinical significance indicates that the magnitude of the difference is substantial enough to be valuable for guiding clinical practice. Both concepts hold their own importance; a statistically significant result may lack clinical significance, and vice versa. Researchers need to consider them from a dialectical perspective.

This study represents the first investigation into optimizing ondansetron OSP dosing for PONV prophylaxis in TJA. The study’s limitations include the following: (1) inadequate statistical power to detect differences below 30% (original sample size calculation targeted larger effect sizes), resulting in non-significant outcomes for some endpoints; (2) suboptimal temporal resolution in endpoint assessment (e.g., lack of hourly PONV monitoring during critical windows such as 7–10 h and 10–13 h postoperatively); and (3) the study did not assess patient satisfaction, quality of recovery, or functional outcomes, which are particularly relevant in orthopedic surgery and could enhance the clinical relevance of the findings. Future studies will further refine the experimental protocol, incorporate larger sample sizes, and employ higher-frequency data collection to delineate dynamic risk patterns and enhance dosing strategies.

## Conclusion

Ondansetron OSPs provide effective and safe prophylaxis against PONV following TJA. Compared to the 8 + 8 + 8 mg regimen, both the Preop 16 mg and 16 + 8 mg strategies demonstrated superior efficacy in reducing PONV incidence. For high-risk patients, the 16 + 8 mg regimen may represent the optimal dosing regimen.

## Data Availability

The original contributions presented in the study are included in the article/supplementary material, further inquiries can be directed to the corresponding authors.
